# Integrated rumen microbiome and serum metabolome analysis responses to feed type that contribution to meat quality in lambs

**DOI:** 10.1186/s42523-023-00288-y

**Published:** 2023-12-19

**Authors:** Shuai Du, Zhenkun Bu, Sihan You, Zipeng Jiang, Weifa Su, Tenghao Wang, Yushan Jia

**Affiliations:** 1 Key Laboratory of Forage Cultivation, Processing and High Efficient Utilization, Ministry of Agriculture, Key Laboratory of Grassland Resources, Ministry of Education, College of Grassland, Resources and Environment, Inner Mongolia Agricultural University, Huhhot, 010019 Inner Mongolia China; 2 Guangdong Laboratory of Lingnan Modern Agriculture, Genome Analysis Laboratory of the Ministry of Agriculture and Rural Affairs, Agriculture Genomics Institute, Chinese Academy of Agricultural Sciences, Shenzhen, 518120 China; 3 National Engineering Laboratory of Biological Feed Safety and Pollution Prevention and Control, Key Laboratory of Molecular Nutrition, Ministry of Education, Key Laboratory of Animal Nutrition and Feed, Ministry of Agriculture and Rural Affairs, Key Laboratory of Animal Nutrition and Feed Science of Zhejiang Province, Institute of Feed Science, Zhejiang University, Hangzhou, 310058 Zhejiang China; 4 Zhejiang Qinglian Food Co., Ltd., Jiaxing, 314399 China

**Keywords:** Grass diet, Concentrate diet, Rumen, Microbiome, Metabolome, Fatty acid profile

## Abstract

**Background:**

Lifestyle factors, such as diet, are known to be a driver on the meat quality, rumen microbiome and serum metabolites. Rumen microbiome metabolites may be important for host health, the correlation between rumen microbiome and production of rumen metabolites are reported, while the impact of rumen microbiome on the serum metabolome and fatty acid of meat are still unclear. This study was designed to explore the rumen microbiome, serum metabolome and fatty acid of meat in response to the grass diet and concentrate diet to lambs, and the relationship of which also investigated.

**Methods:**

In the present study, 12 lambs were randomly divided into two groups: a grass diet (G) and a concentrate diet (C). Here, multiple physicochemical analyses combined with 16S rRNA gene sequences and metabolome analysis was performed to reveal the changes that in response to feed types.

**Results:**

The concentrate diet could improve the growth performance of lambs compared to that fed with the grass diet. The microbiome composition was highly individual, compared to the concentrate group, the abundance of *Rikenellaceae_RC9_gut_group*, *F082_unclassified*, *Muribaculaceae_unclassified*, *Ruminococcaceae_NK4A214_group*, *Bacteroidetes_unclassified*, and *Bacteroidales_UCG-001_unclassified* were significantly (*P* < 0.05) lower in the grass group, while, the abundance of *Succinivibrio*, *Succinivibrionaceae_UCG-002*, *Fibrobacter* and *Christensenellaceae_R-7_group* were significantly (*P* < 0.05) higher in the grass group. Serum metabolomics analysis combined with enrichment analysis revealed that serum metabolites were influenced by feed type as well as the metabolic pathway, and significantly affected serum metabolites involved in amino acids, peptides, and analogues, bile acids, alcohols and derivatives, linoleic acids derivatives, fatty acids and conjugates. Most of the amino acids, peptides, and analogues metabolites were positively associated with the fatty acid contents. Among the bile acids, alcohols and derivatives metabolites, glycocholic was positively associated with all fatty acid contents, except C18:0, while 25-Hydroxycholesterol and lithocholic acid metabolites were negatively associated with most of the fatty acid contents.

**Conclusion:**

Correlation analysis of the association of microbiome with metabolite features, metabolite features with fatty acid provides us with comprehensive understanding of the composition and function of microbial communities. Associations between utilization or production were widely identified among affected microbiome, metabolites and fatty acid, and these findings will contribute to the direction of future research in lamb.

**Supplementary Information:**

The online version contains supplementary material available at 10.1186/s42523-023-00288-y.

## Background

In modern ruminant production system, to achieve better growth performance, carcass traits and meat quality, the pellets and concentrate diet were widely used. Prior research found that the pelleted native grass diet could improve the meat quality and pelleted native grass with concentrate diet can enhances animal performance of lambs [1] The rumen itself is a complex assemblage of bacterial, fungal, archaeal, viral and protozoal microorganisms whose intricate composition and function, which ferments the feed and converts fibrous-rich plant materials and nonhuman edible plant materials to the protein via the rumen microbiome [4]. Additionally, the ruminants could provide an abundant source of the animal protein products to meet the nutrition demand of the growing population worldwide [2, 3]. The rumen is a highly stable and incredibly complicated micro-ecosystem and the rumen microbiome is composed of bacteria, protozoa, archaea, and fungi [5]. This unique microbial ecosystem leads to the development of mutualistic symbiosis between hosts and rumen microbial community composition, which could provide about 70% energy for the ruminant needs [6, 7]. Additionally, the rumen community composition has been linked to host feed efficiency [8], and animal performance [9].

Most interestingly, bacteria play important roles in most of the feed biopolymer degradation and fermentation, which indicated that the bacteria are key players to the host than others [2, 10, 11]. According to a previous report, the rumen community composition and function is strongly influenced by diet, individual genetics and animal age and others [12]. Nevertheless, among these factors, the alternations of the rumen community compositions and functions were determined by the diet [13–15]. Prior researches have indicated that grain- or grass- based feeding animals with a lower bacterial diversity compared to concentrate-rich diet [2, 16], and high forage diets are beneficial for some microorganisms, such as the *Firmicutes* and *Proteobacteria* [17]. Besides, the rumen microbiome is a key component that could communicate with the host via various reservoirs of metabolites [18, 19], and could directly impact the serum metabolome [20]. Therefore, characterizing, quantifying, and understanding the rumen microbial compositions will offer new insights into microbially-mediated metabolic pathway and help to improve feed efficiency and effectiveness [21]. Such insights are of significant scientific, economic, and environmental interest to support modern husbandry.

Accordingly, certain metabolites produced by the microbiome with diet nutrients to influence body metabolism pathway, including the brain-gut axis, gut-liver axis, and/or other pathways [22]. Serum metabolites could directly impact the meat quality of the livestock and the rumen microbiome is important for the fatty acid profiles of meat [23, 24]. Recently published reports found that rumen microbiome and serum metabolites have close relationships, the concentration of serum triglyceride were negatively associated with the genus *Rikenellaceae_RC9_gut_groups* and *Acinetobacter* [23, 25]. Previous report has shown that diet significantly affects the serum metabolites and meat quality of sheep [26]. Notwithstanding, the relationships among rumen microbiome, serum metabolites, and meat quality in lamb fed with native grass without or with concentrate diets remain elusive. Additionally, there is limited information on the differences in microbial compositions and metabolites between grass- or concentrate- fed lambs, after a stable period to adapt the changes diet. It is believed that diet influence the rumen microbiome and serum metabolome, but this has not been well studied in grass- or concentrate-fed lambs on the Mongolian Plateau. The application of 16S rRNA sequencing technology and metabolome to analyze the changes of rumen microbiome and serum metabolites under different feed types is worthy of in-depth exploration. Therefore, we characterized the lamb ruminal fluid microbiome, serum metabolites, and fatty acid profiles of meat to determine the effect of feed types on the ruminal microbiota, metabolites, and meat quality in lamb through a combination analysis of the 16S rRNA gene sequencing and liquid chromatography-mass spectrometry (LC–MS). Finally, the possible relationships among ruminal microbiome, serum metabolites, and fatty acid of meat were also explored.

## Results

### Animal performance

The dry matter intake (DMI) and average daily gain (ADG) of the lambs are presented in Table 1. As expected, no significant (*P* > 0.05) difference was observed at the start of the experimental period in the initial bodyweight between the two groups. At the end of the experiment, the final bodyweight in the concentrate group was significantly (*P* < 0.05) higher than that of the grass group. Additionally, compared to the grass group, the DMI, and ADG of the concentrate were significantly (*P* < 0.05) increased.

**Table 1:** Intake and growth performance of lambs between grass and concentrate groups

Item	G	C	SEM	*P* value
Initial live BW (kg)	27.43	27.36	0.51	0.9476
Final live BW (kg)	32.00b	35.36a	0.75	0.0174
Dry matter intake (kg)	1.58b	1.65a	0.01	0.0043
Average daily gain (g/d)	76.19b	133.33a	11.38	0.0057

G, grass group; C, concentrate group. Means with unlike letters within a row differ at *P* < 0.05; SEM, standard error of the mean

### Rumen microbiome diversity analysis

A total of 886,207 valid reads were obtained, with an average of 73,851 sequences for each rumen sample (data are not shown). Additional file 1: Fig. S1 shows that the numbers of OTUs increased with the sequencing depth. As listed in Table 2, compared to the grass group, the alpha diversity results indicated that the concentrated group decreased the OTUs and Chao1 index, and increased the Shannon index, while no significant (*P* > 0.05) difference was observed between the two groups. Good’s coverage index was higher than 99% in all samples, indicating the accuracy and reproducibility of the sequencing and adequate sequencing depth to investigate the dominant bacterial populations.

**Table 2:** Diversity indices of ruminal microbiome of lambs between grass and concentrate groups

Items	G	C	SEM	*P* value
NO. of operational taxonomic units (OTUs)	1193	1161	35.98	0.6826
Chao1 index	1198.29	1165.45	36.30	0.6725
Shannon index	7.95	8.31	0.16	0.2804
Simpson index	0.98	0.98	< 0.01	0.5490
Good’s coverage index (%)	99.94	99.93	0.01	0.5321

G, grass group; C, concentrate group. Means with unlike letters within a row differ at *P* < 0.05; SEM, standard error of the mean

**Fig. 1:**
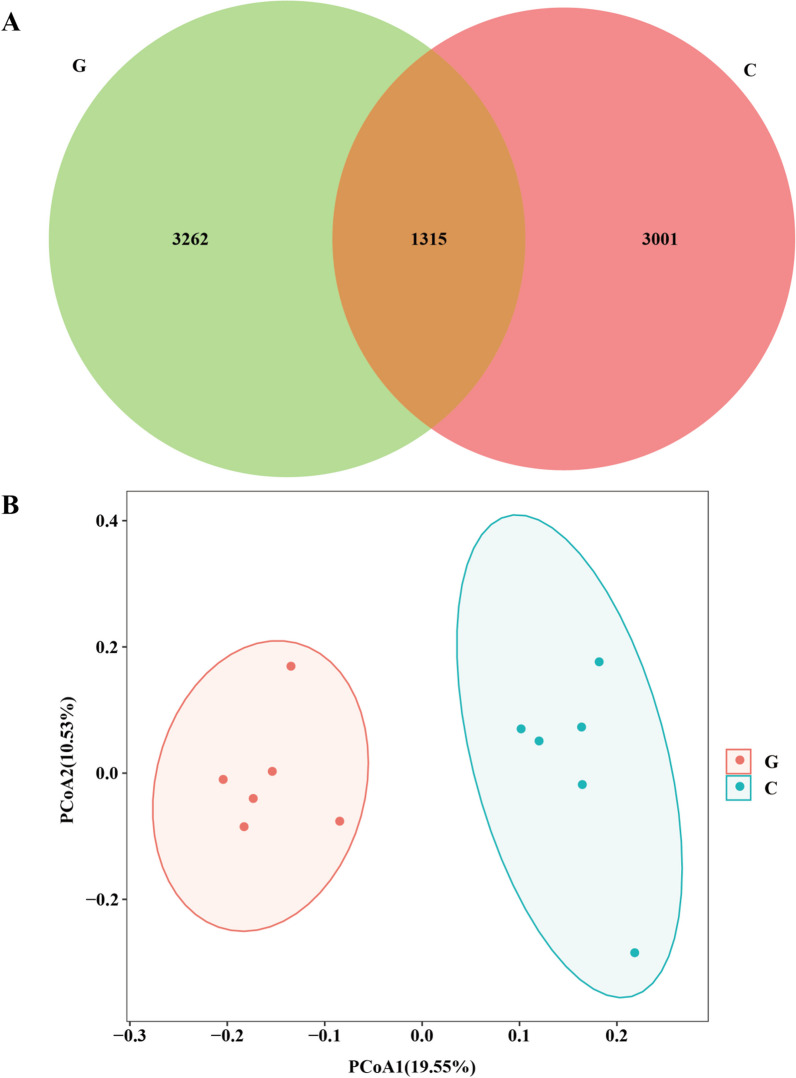
Microbial community among different treatments (n = 6). **A** Venn diagram representing the common and unique operational taxonomic units (OTUs) found at each treatment. **B** Principal coordinates analysis (PCoA) of samples conducted based on unweighted UniFrac distance. Analysis of molecular variance (AMOVA) results is: G versus C (*P* = 0.002). G, grass group; C, concentrate group

In addition, the Venn diagram in the rumen samples showed that the groups shared 1315 OTUs, while the grass and concentrate groups had 3262 and 3001 exclusive OTUs, respectively (Fig. 1A).

To address the effects of feed type on beta diversity, unweighted UniFrac distance was used to characterize the bacterial community across all ruminal samples (Fig. 1B). The principal coordinates analysis (PCoA) profile displayed that the composition of the bacterial community of the grass and the concentrate groups were distinctly separated from each other. Additionally, the analysis of molecular variance result also has a statistically significant difference in the microbiome between the two groups.

### Rumen microbiome composition

Taxonomic analysis of the reads revealed the presence of 425 genera belonging to 26 phyla. At the phylum level, 6 phyla were shown (relative abundance > 1% at least in one group) in Table 3. The most abundant phylum was *Bacteroideters* (43.53% vs. 59.25%), followed by *Firmicutes* (25.12% vs. 25.49%), *Proteobacteria* (20.27% vs. 4.88%), *Kiritimatiellaeota* (3.44% vs. 2.89%), *Fibrobacteres* (2.32% vs. 1.48%) and *Spirochaetes* (1.58% vs. 1.66%). The abundance of *Bacteroideters* was significantly (*P* < 0.05) increased in the concentrate group, the abundance of *Proteobacteria* and *Fibrobacteres* was significantly (*P* < 0.05) higher in the grass group.

**Table 3:** The relative abundance (%) of bacterial phyla (1% at least in one group) of ruminal microbiome of lambs between grass and concentrate groups

Items	G	C	SEM	*P* value
*Bacteroidetes*	43.52b	59.25a	3.74	0.0267
*Firmicutes*	25.12	25.49	1.48	0.9075
*Proteobacteria*	20.27a	4.88b	3.20	0.0075
*Kiritimatiellaeota*	3.44	2.89	0.69	0.7096
*Fibrobacteres*	2.32a	1.48b	0.19	0.0209
*Spirochaetes*	1.58	1.66	0.18	0.8304

G, grass group; C, concentrate group. Means with unlike letters within a row differ at *P* < 0.05; SEM, standard error of the mean

At the genus level, 20 genera were (relative abundance > 1% at least in one group) shown in Table 4. The main genera included *Rikenellaceae_RC9_gut_group*, *Prevotella_1*, *F082_unclassified*, *Succinivibrio*, *and Succinivibrionaceae_UCG-002*. Compared to the concentrate group, the abundance of *Rikenellaceae_RC9_gut_group*, *F082_unclassified*, *Muribaculaceae_unclassified*, *Ruminococcaceae_NK4A214_group*, *Bacteroidetes_unclassified*, and *Bacteroidales_UCG-001_unclassified* were significantly (*P* < 0.05) lower in the grass group. On the other hand, the abundance of *Succinivibrio*, *Succinivibrionaceae_UCG-002*, *Fibrobacter* and *Christensenellaceae_R-7_group* were significantly (*P* < 0.05) higher in the grass group.

**Table 4:** The relative abundance (%) of bacterial genera (1% at least in one group) of ruminal microbiome of lambs between grass and concentrate groups

Items	G	C	SEM	*P* value
*Rikenellaceae_RC9_gut_group*	8.64b	14.41a	1.22	0.1135
*Prevotella_1*	14.00	8.64	1.54	0.0792
*F082_unclassified*	5.54b	13.70a	1.83	0.0168
*Succinivibrio*	9.70a	0.98b	2.86	< 0.0001
*Succinivibrionaceae_UCG-002*	8.51a	0.57b	1.96	0.0350
*WCHB1-41_unclassified*	3.44	2.89	0.69	0.7096
*Bacteroidales_RF16_group_unclassified*	3.12	2.66	0.64	0.7368
*Prevotellaceae_UCG-003*	2.35	3.30	0.36	0.2064
*Muribaculaceae_unclassified*	0.76b	3.32a	0.58	0.0193
*Erysipelotrichaceae_UCG-004*	2.29	1.68	0.38	0.4405
*Prevotellaceae_UCG-001*	1.74	1.91	0.26	0.7595
*Fibrobacter*	2.29a	1.37b	0.19	0.0089
*Christensenellaceae_R-7_group*	2.13a	1.13b	0.26	0.0475
*Succiniclasticum*	1.89	1.35	0.22	0.2398
*Ruminococcaceae_NK4A214_group*	0.96b	2.05a	0.25	0.0177
*Bacteroidales_BS11_gut_group_unclassified*	1.45	1.38	0.4	0.9337
*Bacteroidetes_unclassified*	0.84b	1.72a	0.2	0.0192
*Prevotella*	1.17	1.36	0.17	0.6144
*unclassified*	1.00	1.46	0.25	0.3809
*Bacteroidales_UCG-001_unclassified*	0.83b	1.56a	0.18	0.0421

G, grass group; C, concentrate group. Means with unlike letters within a row differ at *P* < 0.05; SEM, standard error of the mean

**Fig. 2:**
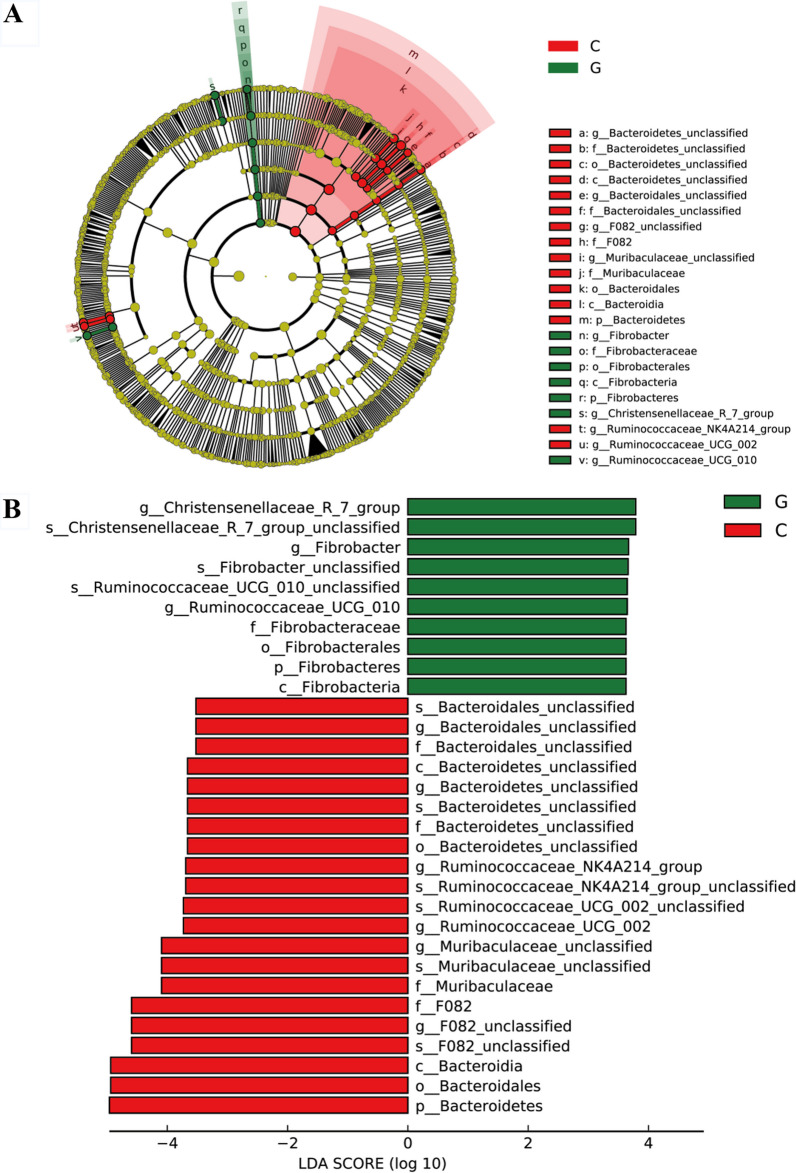
Linear discrimination analysis (LDA) coupled with effect size (LEfSe) analysis of the rumen microbial community of lamb between grass and concentrate groups. **A** Cladogram showing microbial species with significant differences among the two treatment groups. Red, and green represent different groups. Species classification at the phylum, class, order, family and genus level are displayed from inner to outer layers. The red and green and blue nodes represent microbial species in the phylogenetic tree that play important roles in the grass and concentrate groups, respectively. Yellow nodes represent no significant difference between species. **B** Significantly different species with an LDA score greater than the estimated value (default score = 3.5). The length of the histogram represents the LDA score of different species in the two groups. G, grass group; C, concentrate group

As shown in Fig. 2, linear discrimination analysis (LDA) coupled with effect size (LEfSe) analysis revealed the difference in rumen microbiome between the two groups. Figure 2A, B show the differences in the microbiome at various taxonomic levels with LDA scores. Specially, at the genus level, *Fibrobacteraceae*, *Christensenellaceae_R_7_group*, and *Ruminococcaceae_UCG_010* were enriched in the grass group, while, *Bacteroidates_unclassfied*, *F082_unclassified*, *Muribaculaceae_unclassified*, *Ruminococcaceae_NK4A214_group*, and *Ruminococcaceae_UCG_002* were enriched in the concentrate group.

### Serum metabolome profiling

**Fig. 3:**
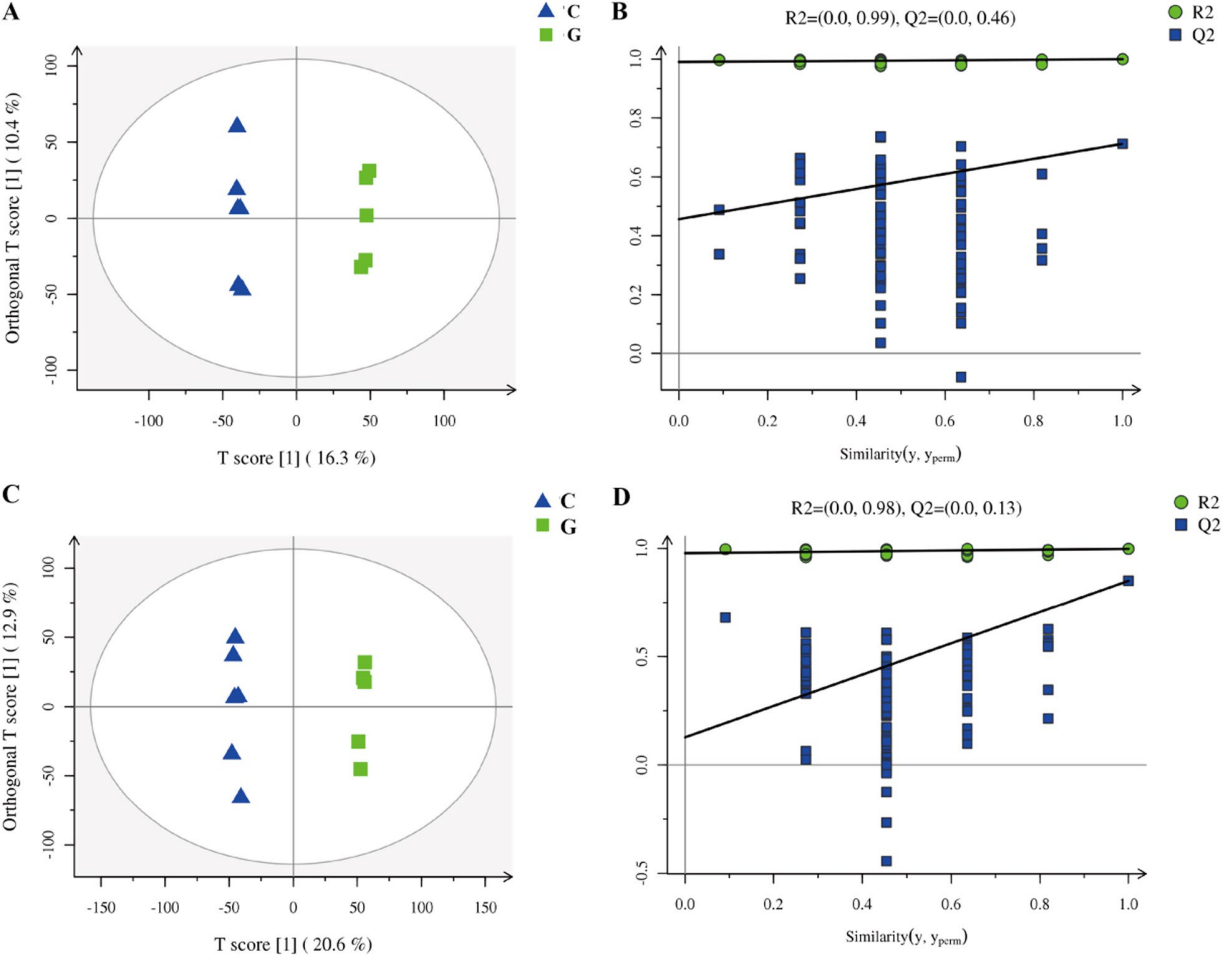
Orthogonal partial least squares discriminant analysis [(O)PLS-DA] plot of lamb rumen metabolites in comparisons of the concentrate and grass groups following (**A**, **B**) positive and (**C**, **D**) negative mode ionization

To assess the functional profile of the rumen microbiome of feed types, untargeted LC–MS analysis on all serum samples (1 sample per lamb, collected before slaughter). In the present study, all data, including the QC samples that were included throughout the analysis, were first examined by principal component analysis (PCA) following positive (Additional file 1: Fig. S2A) and negative mode ionization (Additional file 1: Fig. S2B) to provide a total overview of the differences among the metabolome. Score plots of the (O)PLS-DA carried out to display the different metabolites between the two groups and supervise the multivariate analysis are shown in Fig. 3. The (O)PLS-DA provides valuable insights into group relationships from simple visual inspection of scores-space clustering patterns. All the samples in the score plots were within the 95% Hotelling T2 ellipse. For the positive ionization analysis, the (O)PLS-DA fitted model (Fig. 3A) resulted in one predictive and two orthogonal components. Furthermore, 26.7% of the total explained variation in the data set (R^2^X cum) was used to account for 99.9% of the variance in the class separation (R^2^Y cum), and the cross-validated predictive ability of the model was 67.4% (Q^2^ cum). As shown in Fig. 3B, the permutation test (R^2^Y = 0.99, Q^2^Y = 0.46) indicated that the model was adequate for its efficacy. The results of the (O)PLS-DA results and permutation tests following negative mode ionization are shown in Fig. 3C, D Both positive and negative data revealed clear separation and discrimination between the concentrate and grass groups, illustrating the effectiveness of the (O)PLS-DA model can be used to identify different metabolites between the two groups.

Overall, a total of 290 compounds were identified in the serum metabolome. After *t*-test and variable importance in projection (VIP) filtering for the relative contents of serum, 54 metabolites were significantly different between the two groups, 35 of these metabolites were positively ionized metabolites (Additional file 1: Table S2) and 19 of these metabolites were negatively ionized metabolites (Additional file 1: Table S3). Among 35 positive ionized metabolites, 10 were classified as amino acids, peptides, and analogues, 2 as bile acids, alcohols and derivatives, 2 as purines and purine derivatives, 2 as phosphate esters. In the negative ionization analysis, 19 differential metabolites were classified as amino acids, peptides, and analogues, benzoic acids and derivatives, lineolic acids derivatives and fatty acids and conjugates. The comparison analysis revealed that the relative concentrations of 40 metabolites were significantly higher in the serum of the grass group, and the relative concentrations of 14 metabolites were significantly higher in the serum of the concentrate group (*P* < 0.05, VIP > 1).

**Fig. 4:**
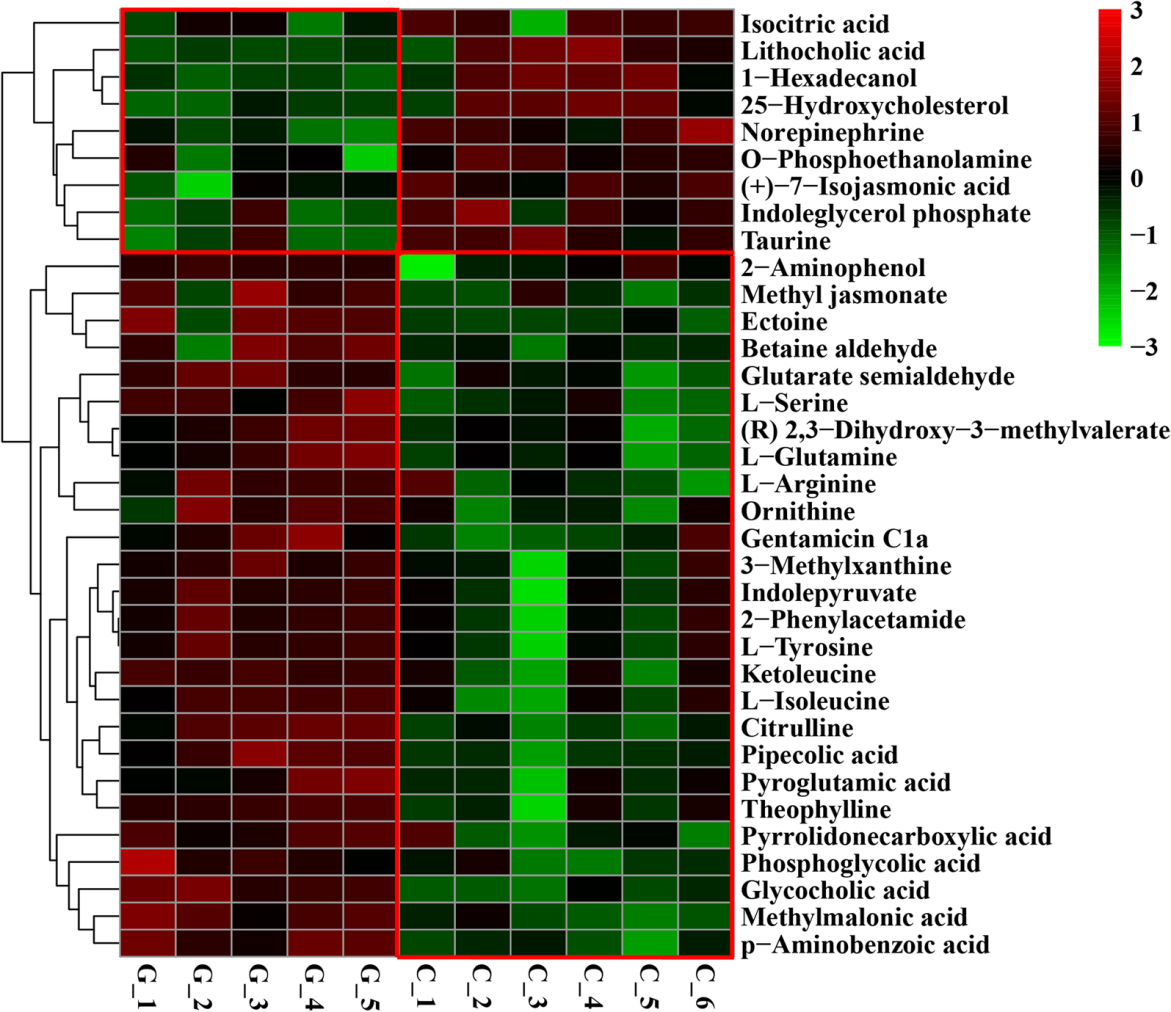
Hierarchical clustering analysis for identification of different metabolites in lamb serum by comparison of the grass and concentrate groups following positive mode ionization. Each column in the figure represents a sample, each row represents a metabolite, and the color indicates the relative amount of metabolites expressed in the group; Red indicates that the metabolite is expressed at high levels, and green indicates lower expression

**Fig. 5:**
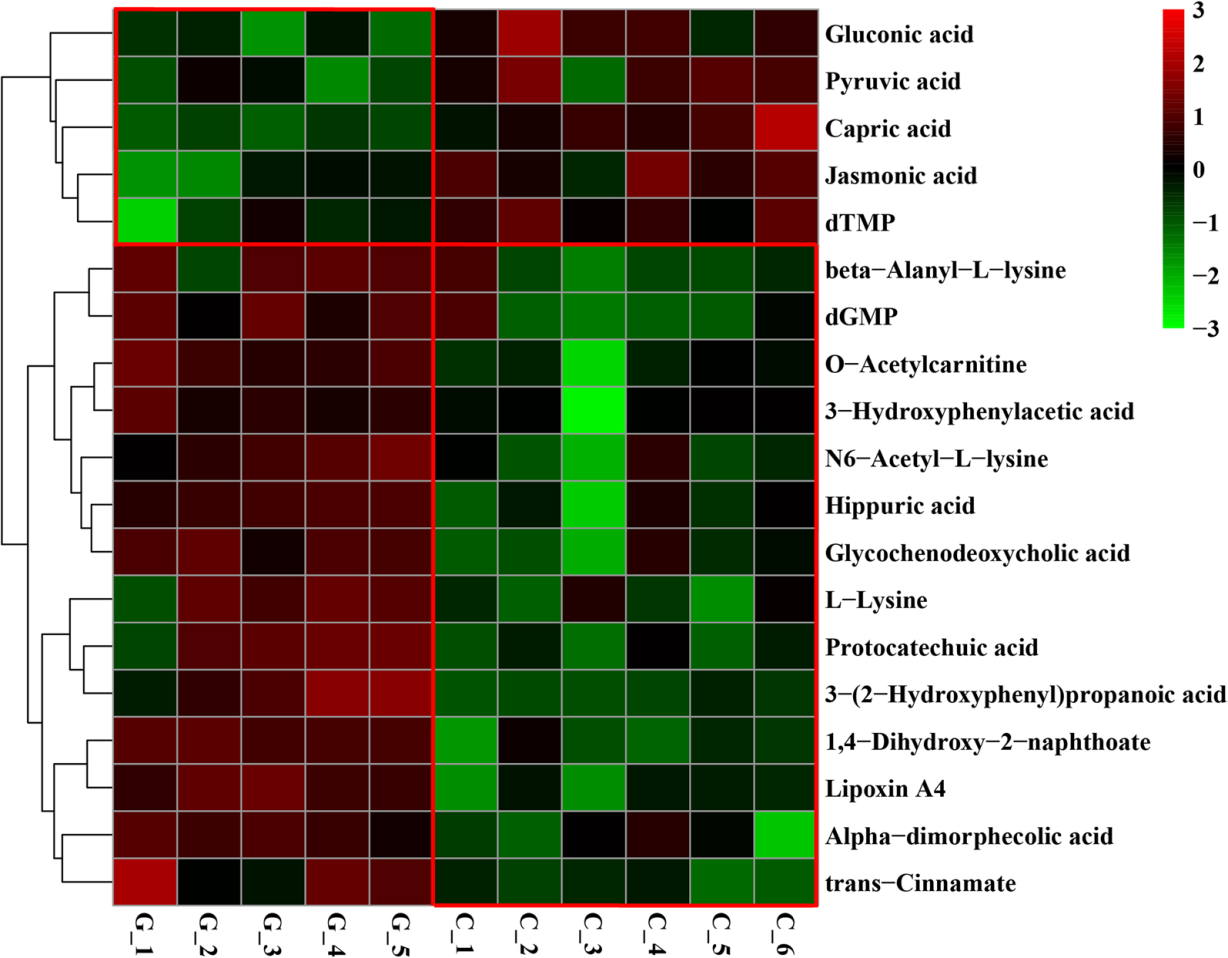
Hierarchical clustering analysis for identification of different metabolites in lamb serum by comparison of the grass and concentrate groups following negative mode ionization. Each column in the figure represents a sample, each row represents a metabolite, and the color indicates the relative amount of metabolites expressed in the group; Red indicates that the metabolite is expressed at high levels, and green indicates lower expression

To visualize the differences in the lamb rumen metabolome associated with the feed type, the hierachical clustering analysis (HCA) with a heat map was performed. For the positive ionization data (Fig. 4), two distinct clusters were formed among these differential metabolites. It was observed that 9 metabolites were expressed at a lower level in the grass group, including (+)-7-isojasmonic acid, 1-hexadecanol, 25-hydroxycholesterol, indoleglycerol phosphate, isocitric acid, lithocholic acid, norepinephrine, *O*-phosphoethanolamine and taurine, and 26 metabolites were expressed at a lower level in the concentrate group, including (R) 2,3-dihydroxy-3-methylvalerate, 2-aminophenol, 2-phenylacetamide, 3-methylxanthine, citrulline, ectoine, gentamicin c1a, glutarate semialdehyde, glycocholic acid, indolepyruvate, ketoleucine, l-arginine, l-glutamine, l-isoleucine, l-serine, l-tyrosine, methyl jasmonate, methylmalonic acid, ornithine, p-aminobenzoic acid, phosphoglycolic acid, pipecolic acid, pyroglutamic acid, pyrrolidonecarboxylic acid, theophylline and betaine aldehyde. For the negative ionization data (Fig. 5), two distinct clusters were also formed among these differential metabolites. It was observed that 5 metabolites were expressed at a lower level in the grass group, including gluconic acid, pyruvic acid, capric acid, jasmonic acid and dTMP, and 14 metabolites were expressed at a lower level in the concentrate group, including beta-alanyl-l-lysine, dGMP, O-acetylcarnitine, 3-hydroxyphenylacetic acid, *N*6-acetyl-l-lysine, hippuric acid, glycochenodeoxycholic acid, l-lysine, protocatechuic acid, 3-(2-Hydroxyphenyl) propanoic acid, 1,4-dihydroxy-2-naphthoate, lipoxin A4, Alpha-dimorphecolic acid and trans-cinnamate. The feed type had a significant effect on the serum metabolome and such differences were clearly observed in the clusters generated in the heatmap plot generated by HCA.

**Fig. 6:**
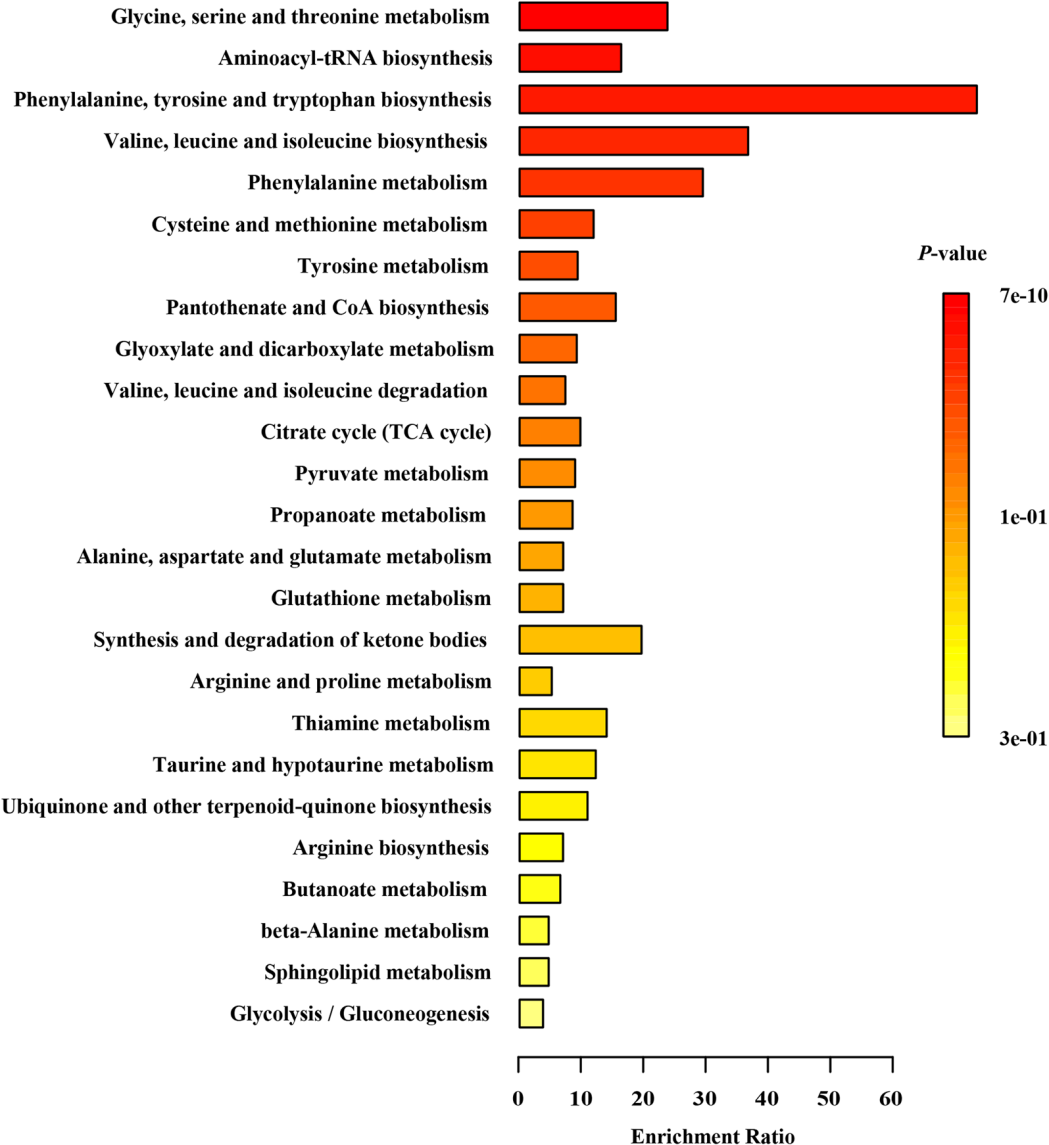
Metabolic pathway enrichment analysis based on kyoto encyclopedia of genes and genomes (KEGG) database

Pathway topology analysis was carried out according to the metabolites identified. The four enriched major metabolic pathways between the grass and concentrate groups have been shown in Fig. 6, including glycine, serine and threonine metabolism, aminoacyl-tRNA biosynthesis, phenylalanine, tyrosine and tryptophan biosynthesis, valine, leucine and isoleucine biosynthesis, phenylalanine metabolism, and others.

### Correlations between rumen bacteria and serum metabolome

**Fig. 7:**
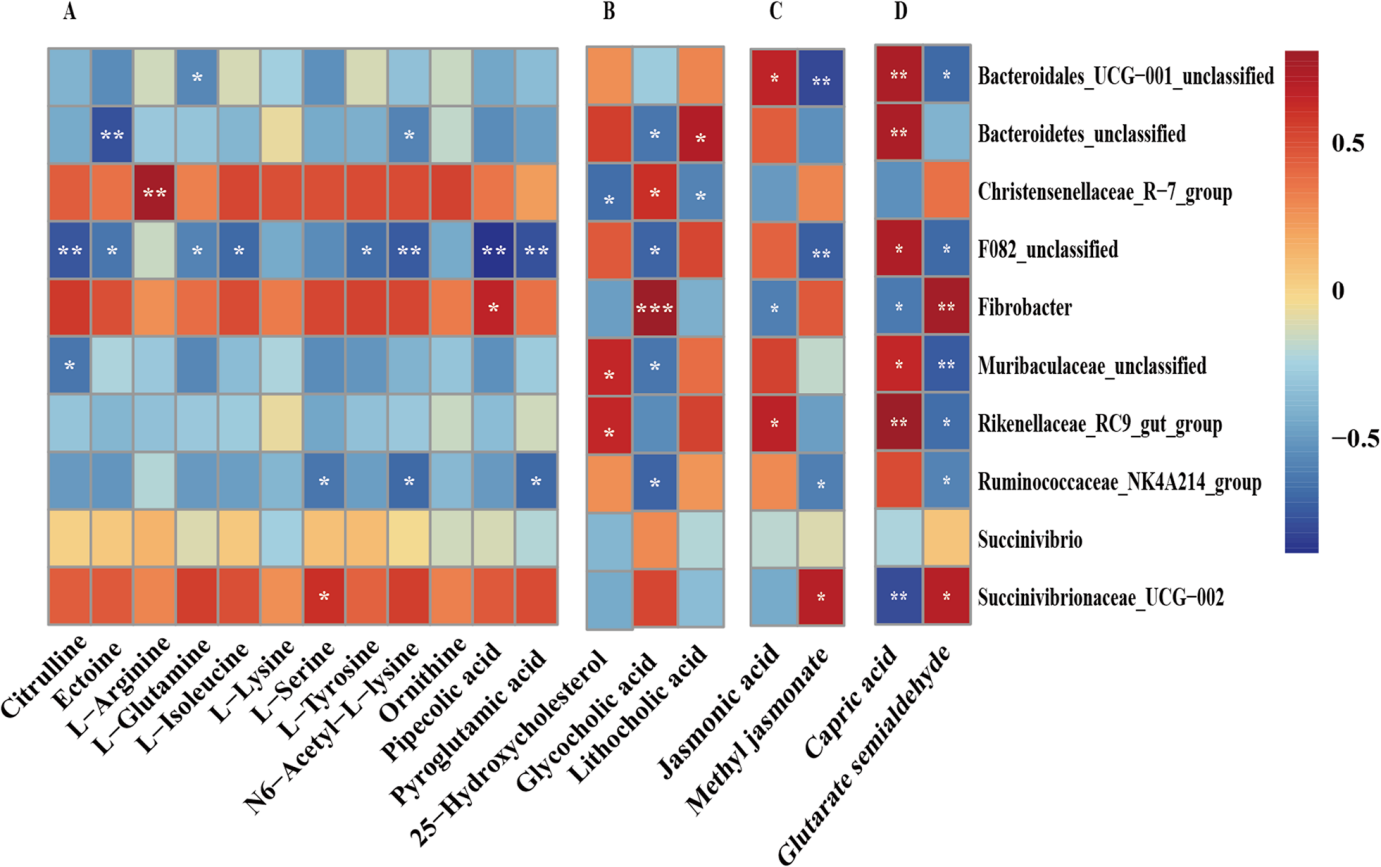
Correlation analysis between genera (top 20 significant genera) and metabolite concentrations (based on VIP and *P* value) affected by the feed type. **A** Amino acids, peptides, and analogues. **B** Bile acids, alcohols and derivatives. **C** Linoleic acids derivatives. **D** Fatty acids and conjugates. Each row in the graph represents a genus, each column represents a metabolite, and each lattice represents a Person correlation coefficient between a component and a metabolite. Red represents a positive correlation, while blue represents a negative correlation. *Significant correlation between the grass group and concentrate group (*P* < 0.05), **Significant correlation between the grass group and concentrate group (*P* < 0.01), ***significant correlation between the grass group and concentrate group (*P* < 0.001)

The correlation between rumen bacteria and major metabolites was characterized and shown in Fig. 7, the metabolites including amino acids, peptides, and analogues, bile acids, alcohols and derivatives, linoleic acids derivatives and fatty acids and conjugates. In the amino acids, peptides, and analogues, the results of the present study showed that the genus *Bacreroidates unclassfied* was negatively associated with ectoine, while *Christensenellaceae R-7 group*, *Fibrobacter* and *Succinivibrionaceae UCG-002* were positively associated with l-arginine, ornithine and l-serine, respectively. In the bile acids, alcohols and derivatives, *Fibrobacter* was positively associated with glycocholic. The correlations between rumen bacteria and linoleic acids derivatives metabolites, between rumen bacteria and fatty acids and conjugates are various.

### Meat fatty acid profile

The fatty acid compositions of meat from Ujumqin lambs in the grass and concentrate groups are shown in Table 5. The C14:0, C14:1, C15:0, C15:1, C16:0, C16:1, C17:0, C17:1, C18:0, C18:2n6t, C20:0, C21:0 and C22:0 contents were significantly (*P* < 0.05) lower in the grass groups than in the concentrate group. The C18:3n3 and C22:6n3 contents were significantly (*P* < 0.05) higher in the grass group than in the concentrate group.

**Table 5:** Fatty acid of *Longissimus lumborum* muscle of Ujimqin lambs between grass and concentrate groups (mg/100 g)

Items	G	C	SEM	*P* value
C14:0	70.83b	116.93a	7.37	< 0.0001
C14:1	6.54b	10.30a	0.74	0.0038
C15:0	12.40b	22.57a	1.95	0.0025
C15:1	3.95b	8.84a	0.86	0.0004
C16:0	811.33b	1085.00a	53.30	0.0100
C16:1	53.33b	76.23a	4.53	0.0040
C17:0	35.70b	63.17a	5.18	0.0018
C17:1	27.53b	38.80a	2.17	0.0025
C18:0	666.67b	942.00a	54.99	0.0113
C18:1n9t	75.57	88.27	6.14	0.3240
C18:1n9c	1360.00	1420.00	33.94	0.4024
C18:2n6t	8.42b	12.93a	0.73	< 0.0001
C18:2n6c	144.33	149.67	5.25	0.6437
C20:0	10.57b	16.80a	0.96	< 0.0001
C18:3n3	38.77a	17.97b	3.30	< 0.0001
C21:0	17.70b	25.90a	1.57	0.0023
C22:0	14.37b	17.57a	0.62	0.0029
C20:4n6	42.97	47.23	1.42	0.1405
C24:0	26.05	22.10	2.72	0.4939
C22:6n3	11.87a	10.21b	0.29	0.0002

G, grass group; C, concentrate group. Means with unlike letters within a row differ at *P* < 0.05; SEM = standard error of the mean

### Correlations between fatty acid and serum metabolites

**Fig. 8:**
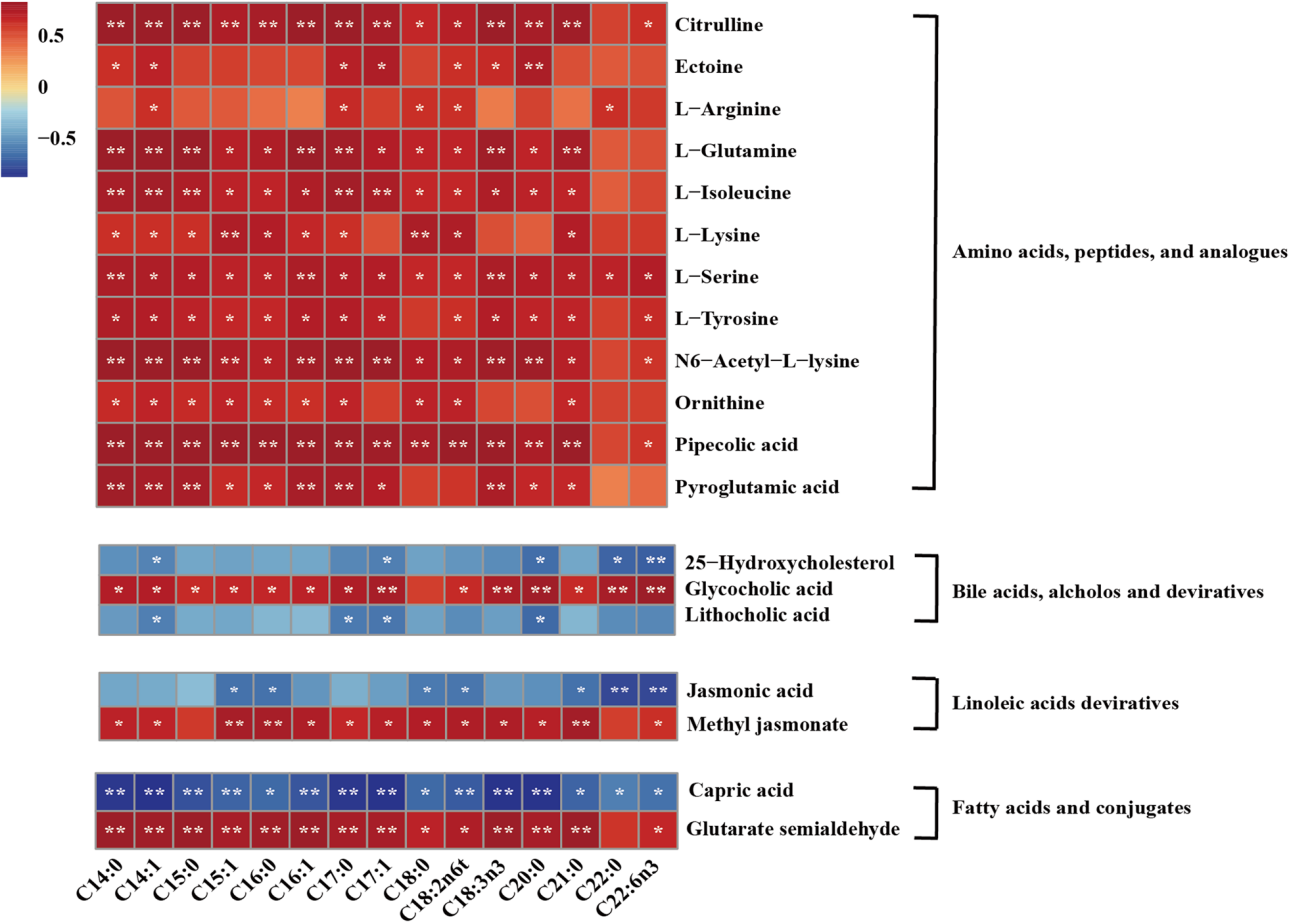
Correlation analysis between metabolite concentrations and fatty acids affected by the feed type. Each row in the graph represents a metabolite, each column represents a fatty acid, and each lattice represents a Person correlation coefficient between a metabolite and a component. Red represents a positive correlation, while blue represents a negative correlation. *significant correlation between the grass group and concentrate group (*P* < 0.05), **significant correlation between the grass group and concentrate group (*P* < 0.01)

The correlation between major metabolites and fatty acid content of lambs was characterized and displayed in Fig. 8. Most of the amino acids, peptides, and analogues metabolites were positively associated with the fatty acid contents. Among the bile acids, alcohols and derivatives metabolites, glycocholic was positively associated with all fatty acid contents, except C18:0, while 25-Hydroxycholesterol and lithocholic acid metabolites were negatively associated with most of the fatty acid contents. In the alcohols and derivatives, linoleic acids derivatives and fatty acids and conjugates metabolites, the correlations between fatty acid and serum metabolites were complex.

### Metabolites and metabolic pathways

**Fig. 9:**
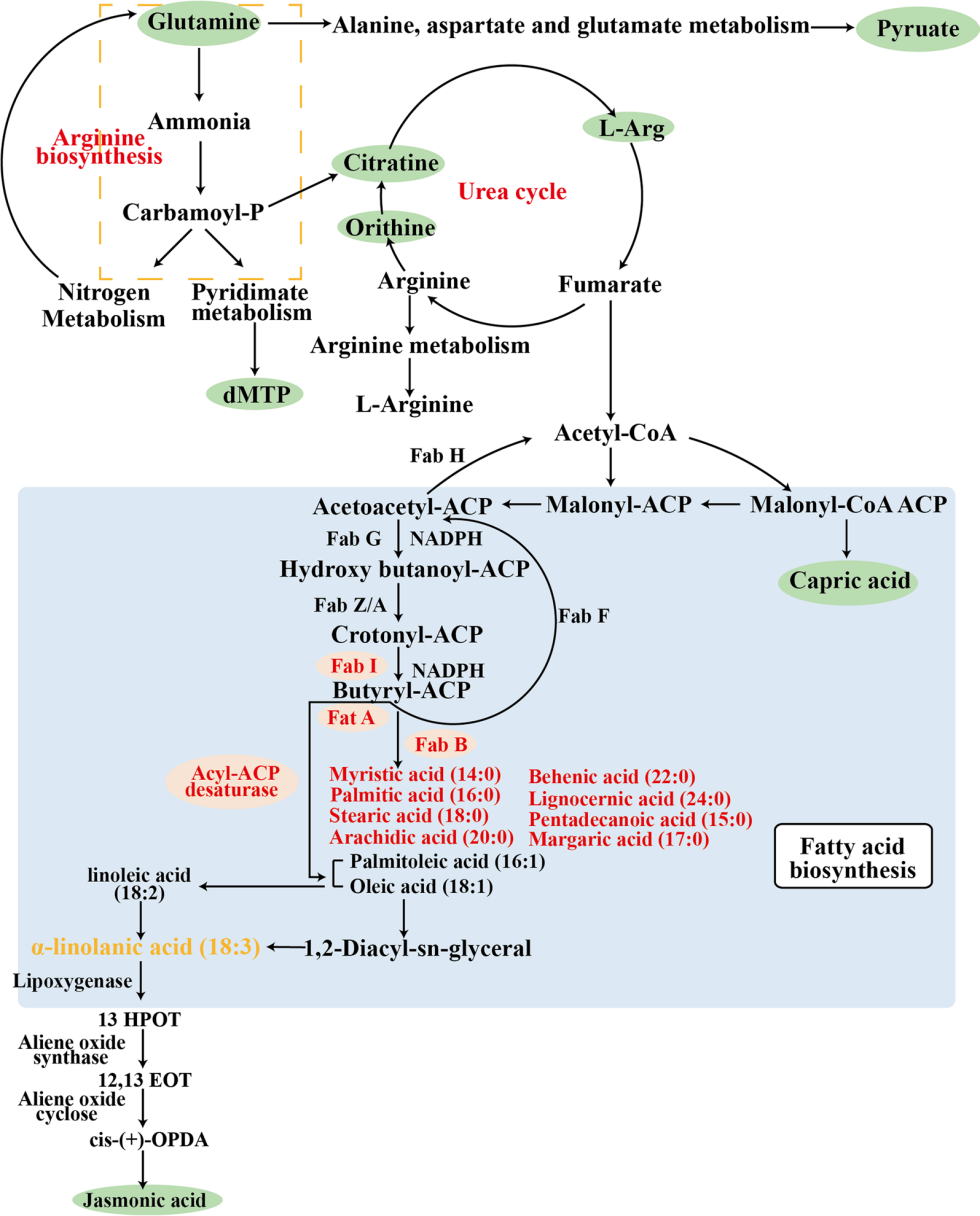
Serum metabolites pathway related to fatty acid in the *Longissimus lumborum* muscle

The metabolites concentrations and related fatty acid contents of meat were visualized as heat maps and used to evaluate the possible biochemical pathways adapted from the kyoto encyclopedia of genes and genomes (KEGG) database (Fig. 9). The metabolome analysis indicated that the metabolites directly influenced the fatty acid synthesis and contents via arginine biosynthesis, urea cycle, alanine, aspirate and glutamate metabolism, arginine and proline metabolism, nitrogen cycle, pyrimidine metabolism, and tricarboxylic acid cycle. The critical metabolites that participated in substance synthesis and conversion through the pathway were indicated by the green area, such as glutamine is the dominant metabolite in arginine biosynthesis, dMTP is associated with pyridimate metabolism, pyruate participates alanine, aspirate and glutamate metabolism, orthine, citratine and l-arginine is the primary metabolites in urea cycle.

## Discussion

There is great interest in the impact of feed type on growth performance and meat quality, in part, mediated via its effects on the composition of the rumen microbiota and serum metabolites. In the present study, we used an integrated approach, combining 16S rRNA sequencing to evaluate the overall ruminal bacterial composition and LC–MS to determine the serum metabolites. This is the first tentative model to associate the signatures of feed type with integrative analysis of the microbiome and metabolome.

In the present study, lambs fed concentrate diet had higher final bodyweight, DMI, and ADG than that in grass group. These results are consistent with Bu et al. and Du et al. who found the concentrate diet could improve the animal performance and increase ADG [27, 28], which could be explained by the lower fiber content and higher energy intake in the concentrate group [12].

Our results suggest that feed type could influence the rumen bacterial community composition in lambs. The higher OTU number and Chao1 index were observed in the grass group compared to the concentrate group. These results were similar to Liu et al. who found the forage group animals with higher bacterial diversity and richness than that in concentrate diet fed animals [21]. No significant differences were observed in Shannon and Simpson indices between the two treatments and the grass group had lower community evenness than the concentrate group, which could be contributed by the increased DMI and higher energy intake in the concentrate that directly influence the bacterial growth rate [29, 30].

The changes in the rumen bacterial compositions were also explored, our results suggest that the grass and concentrate group has its distinct microbiome, as reflected by the clustering of the samples by diet group using PCoA. Macroscopically, the different diets drove a separation in the bacterial community, the distinguishable changes between the two groups, following the reports that noticeable separation of the microbial structure was observed among forage, grain, and concentrate diets [12, 29], which could be a contributed to the growth of microorganisms under various pH conditions [30].

In the present study, the predominant phyla include *Bacteroidetes*, *Firmicutes*, *Proteobacteria*, *Kiritimatiellaeota*, *Fibrobacteres*, and *Spirochaetes*, with accounting for approximately 90% of bacterial species, in agreement with previous findings in lambs [3, 12, 29]. The predominant bacterial phylum, *Bacteroidetes* and *Firmicutes,* is to degrade carbohydrates and proteins that have been reported [2, 31]. The primary role of *Bacteroides* is connected to degrade diverse plant polysaccharides and improves the nutrient utilization of the host to enhance the host’s immunity [32–34]. The abundance of *Bacteroides* in the concentrate diet group was higher compared to that in the grass group, which is followed with Xu et al. and Trabi et al. which could be explained by the degradation of carbohydrates under different feed types [12, 35]. *Firmicutes* is another important role in the degradation of fiber and cellulose, and is associated with the decomposition of polysaccharide and the utilization of energy [36–38]. However, no significant difference was observed in *Firmicutes* in the two groups, sufficient fiber and cellulose may be the main reason. The higher abundance of Firmicutes was found in the concentrate diet fed lamb, which is in accordance with previous reports that the concentrate could enhance the abundance of Firmicutes [39, 40]. It is known that *Proteobacteria* is a biomarker for inflammatory progression and enhances the progression of the disease [41, 42]. The higher abundance of *Proteobacteria* was found in the grass group compared to the concentrate group, which is similar with the previous research that the increased concentrate proportions could increase the abundance of *Proteobacteria* [43]. *Fibrobacter* is a dominant phylum to utilize plant cellulose for ruminants and they are usually found in fiber-rich diets [43, 44]. The abundance of *Fibrobacter* was significantly increased in the grass group compared to the concentrate group. These results indicated that *Fibrobacter is* actively involved in the digestion of fiber and is in agreement with the higher fiber contents in diets [35].

More detailed differences in the microbiome could explain their changes under different feed types. At the genus level, the abundance of *Rikenellaceae RC9 gut group* in the concentrate group was significantly greater compared to the grass group, which was similar to the results of prior reports which found this genus involved in the carbohydrate degradation [35, 45]. The genus *Prevotella* could produce succinate and acetate by utilize proteins and starch in the diet, and is a core genus within the rumen community [46, 47]. The abundance of *Prevotella* 1 at a lower level in the concentrate group, in which similar dynamics were described which found the increased abundance of the *Provotella* in a higher fiber percentage diet [48, 49]. F082 were also decreased in the grass group compared to the concentrate group. These results illustrate that within this genus and uncultured family significant ecological diversity exists to the host and feed type, however, the ruminal role of the *Bacteroidetes* unclassified family F082 is still unclear [50]. The genus *Succinivibrio*, two short-chain fatty acid-producing bacteria [51, 52], was significantly decreased in the concentrate group. These results were similar with the prior reports which found grass-diet increased the abundance of *Succinivibri* [53]. Besides, the abundance of *Succinivibrionaceae UCG-002*, is the main participant to produce propionate [21], was markedly increased in the grass group. These included changes in the *Succinivibrionaceae* family that changed in relative abundance in diet-related changes [14]. Similarly, a higher abundance of *Fibrobacter* was observed in the grass group, in accordance with Fernando et al. [43], which contributes to the genus *Fibrobacter* has a highly ability in degrading cellulose [54]. These results again prove that rumen microbial community could respond to feed type by the changes of nutrient compositions.

LC–MS based metabolome is a widely, efficient and effective used method for determine metabolic status and functional metabolites [57]. The LC–MS based metabolome analysis was used in this study to further understand the effects of feed type on the metabolites. Interestingly, contrary to the minor fluctuations of the rumen microbiome observed above, feed type had large effects on serum metabolites. The PCA and (O)PLS-DA scatter plots displayed significant differences in the serum metabolites between the grass diet and concentrate diet, and showed the obvious effects of diet on serum metabolites. We found that most of the metabolites differed between the grass and concentrate groups belonged to amino acids, peptides, and analogues, bile acids, alcohols and derivatives, benzoic acids and derivatives, purines and purine derivatives, phosphate esters, fatty acids and conjugates involved in distinct metabolic processes. Certain amino acids, peptides, and analogues, bile acids, alcohols and derivatives, fatty acids and conjugates are not only directly influence by the diet, but are also important biomarkers of meat quality traits in animals [58]. Amino acids, peptides, and analogues, which are key precursors for polypeptides and proteins synthesis, are mainly derived by proteins of diet and microproteins [21, 59]. Amino acids, peptides, and analogues related metabolites, such as citrulline, l-glutamine, l-arginine, l-arginine, l-serine, l-isoleucine, l-lysine, l-tyrosine and phosphoglycolic acid consisting of arginine biosynthesis pathway, aminoacyl-tRNA biosynthesis pathway and glyoxylate and dicarboxylate metabolism pathway and the other pathways. The prior report found that l-glutamine is degraded into l-glutamate through hydrolysis mediated by the ruminal microbes, plays an important role in maintaining multiple important functions, which could influence microbial growth and efficiency by acting as a potential inhibitor of the utilization of amino acids, peptides, and analogues by ruminal bacteria such as nutrient metabolism, immune response, and intestinal integrity, as well as the synthesis of other bioactive compounds [21, 60]. In the present study, a part of these results was inconsistent with Zhang et al. who found the metabolites of ornithine [61], and l-tyrosine significantly increased in the concentrate diet, which could be explained by the chemical compositions of the diet [62]. The increase of metabolites of ornithine and l-tyrosine provide available nutrients for the host, but also enhance the potential risk for the intestines [61]. Early work showed that ornithine and l-tyrosine are precursors of putrescine and tyramine, which are generated by microbial activity, benefited for biogenic amines accumulation and maybe increase intestinal pathological processes [63–65]. Whereas, in the present study, the putrescine and tyramine were not detected might be contributed by the limitations of LC–MS method or the concentrations of putrescine and tyramine were lower than then the detected level [61]. Simultaneously, contrary to the alterations in the concentrate group, the grass group decreased the concentrate of isocitric acid, *O*-phosphoethanolamine, taurine and pyruvic acid compared with the concentrate group. Additionally, glycocholic acid, 25-Hydroxycholesterol and lithocholic acid are associated with bile acids, alcohols and derivatives pathway. Bile acids are related to the absorption and metabolism of diet lipids, interact with the rumen microbiome [60, 66]. In the present study, the higher primary bile acid (glycocholic acid) and lower concentrate of secondary bile acids (lithocholic acid) was significantly higher in the grass group. These results might indicate the conversion rate of bile acids from the primary metabolites to the secondary metabolites was decreased in the grass group, which following the result who found lower conversion rate of bile acids from the primary metabolites to the secondary metabolites form with a lower bodyweight [69]. These result also consistent with the lower final bodyweight of lambs in the grass group.

Diet is a critical role in the fat metabolism process [67]. In the present study, the fatty acid contents in the concentrate group were significantly influenced by the diet and various fatty acid contents were higher in the concentrate group than that in the grass group, which is following the prior reports that the concentrate diet could enhance the fatty acid contents [27, 28]. Indeed, the metabolic pathway of fatty acid biosynthesis in sheep can be changed by feed type [67]. Fatty acid contents of meat largely depend on the dietary fatty acid sources, along with other factors like the ruminal bacterial and blood fatty acid synthesis [67, 68]. Glutarate semialdehyde, methyl jasmonate, jasmonic acid and capric acid are associated with linoleic acids derivatives, and fatty acids and conjugates pathways. Rumen microbiome could rapidly hydrogenate fatty acids ingested by the rumen through diet [35]. Before being hydrogenated into a saturated end-product, lipase, galactosidase, and phospholipase were produced by rumen microbes remove unesterified fatty acids and different intermediate fatty acids, especially odd-chain fatty acids [69].

Diet not only influences the rumen microbiome, but host metabolism and meat quality also regulated by the microbiome [59, 60, 70]. Ruminant meat is characterized by having considerable percentages of fatty acids profiles, and these profiles are subjected to a process of biohydrogenation conducted by rumen microbiome [71]. The *Rikenellaceae*_RC9_gut_group was the butyrate-producing bacteria and could increase the AMPK activity to regulate the lipid deposition traits by changes the production of VFAs [72]. Additionally, it has been indicated that *Prevotellaceae*_UCG-003 is efficiently metabolize into fatty acid synthesizing [73, 74]. Therefore, the higher fatty acid profiles were found in the concentrate group. Correlation analysis provided new insights to identify several new bacterial genera potentially implicated in the host metabolism for us. The relationship between the rumen microbiome and metabolome has been investigated on goat [75], the interaction of microbial metabolome and metagenome was also reported on dairy cows [2]. However, whether and how the rumen microbiome could interact with the serum metabolites to response feed type and meat quality remains unknown. Therefore, we identified the correlation among rumen microbiome, serum metabolome and fatty acid profile, and association analysis revealed a correlation between the abundance of specific bacterial genera and metabolites, and between the metabolites and fatty acid contents of meat that were significantly affected by feed type. The characterization of metabolic alterations modulated by the rumen microbiome has been used to understand the molecular mechanisms of host health and disease development in animals [60, 76], and the fatty acid profile of meat was also modulated by the serum metabolites. Altogether, the diet directly regulated the disruption of rumen microbial composition and metabolic homeostasis, and then rumen microbiome and serum could be a major underlying factor that influences growth performance and meat quality of lambs.

## Conclusion

In summary, this study involved a combination of physicochemical analyses, microbiome and metabolome analyses, the associations between the specific bacterial genera and metabolites, and metabolites and fatty acid were significantly influenced by feed type. These results could provide a better understanding of meat quality, serum metabolites and microbial functions that contribute to the development of modern lamb husbandry strategies. Furthermore, the causes and mechanisms driving the interactions among ruminal bacteria, serum metabolism and meat quality merit further investigation.

## Methods

### Animals and experimental design

A total of twelve Ujumqin lambs (6 months, 27.39 ± 0.51 kg) were used in the present study. The experiment was conducted at the Lvye Grass-based Livestock Husbandry Development CO., Ltd, (Xilin Hot, China). Lambs were randomly assigned to two treatments (n = 6) and kept in individual pens (2.0 by 2.0 m). Lambs were assigned to either of two treatments groups based on the composition of the pelleted diet offered: native grass only (G) or native grass (70%) and concentrate (30%) (C). The native grass was harvested from the typical steppe in Xilin Hot, China. The typical steppe was composed by *Stipa gigantea* L., *Leymus chinensis* (Trin.) Tzvel., *Lespedeza davurica* (Laxm.) Schindl, *Allium mongolicum* Regel, *Thalictrum petaloideum* Linn., *Bupleurum chinensis* DC., *Serratula centauroides* Linn., *Caragana microphylla* Lam, and others, the dominant species were *Stipa gigantea* L., and *Leymus chinensis* (Trin.) Tzvel. The pre-experiment lasted for 15 days, and the feeding experiment lasted for 60 days for the data collection. The lambs were fed based on the consumption of 110% of their expected intake at 08:00 and 16:00, the lambs had free access to fresh drinking water. The feed intake was record daily for each pen of lambs by weighing all feed offered during the experimental period and recording the number of daily meals refused. All lambs were weighed before the morning feeding with an empty stomach and without fasting throughout the experimental period in the morning (06:00–07:00 h) and with 7-d intervals, the initial and final bodyweight were also recorded. The body weight gain was calculated as the difference between the final body weight and the initial body weight. The ingredients and compositions of the experimental diets are listed in Additional file 1: Table S1.

### Feed compositions analysis

The feed dry matter (DM) content was determined by drying a sub-sample in an oven for 72 h at 65 °C and then grinding it through a 1 mm screen (FW100, Taisite Instrument Co., Ltd., Tianjin, China) for further chemical analysis. The ANKOM A200i Fiber Analyzer (ANKOM Technology, Macedon, NY, USA) was utilized to determine the fiber compositions, including the neutral detergent fiber (NDF) and acid detergent fiber (ADF) contents, following previous reports [77]. The crude protein (CP) and ether extract (EE) content was determined using the method of the Association of Official Analytical Chemists [78].

### Sample collection

When the experiment is finished, the lambs were transferred and slaughtered at a commercial slaughterhouse after fasting for 24 h. After slaughtering, the rumen content of lamb was first homogenized by hand using disposable polyethylene gloves and the whole rumen contents were strained through four layers of cheesecloth for the rumen fluid samples. Then, approximately 100 mL of rumen samples were placed in sterile centrifuge tubes and immediately frozen in liquid nitrogen containers, then stored at − 80 °C until analysis. Blood samples were taken before transferring to the slaughterhouse by caudal venipuncture using coagulation-promoting (Becton, Dickison and Co., Franklin Lakes, NJ) collected in tubes with the anticoagulant ethylene diamine tetraacetic acid for metabolites analyses. Blood samples were centrifuged within 1 h of collection for 15 min at 3000×*g* and 4 °C, and serum was collected. Serum samples were stored at − 20 °C until analysis. A total of 12 serum samples were collected in this study, unfortunately, 1 serum sample from the concentrate group was damaged and the analysis was performed using the left 11 serum samples. *Longissimus lumborum* muscle was selected for fatty acid profile analysis and collected from the carcass on the right side of the vertebrae, and stored in a freeze at − 20 °C until analysis. All samples were analyzed within a month.

### DNA extraction, 16S rRNA gene amplification and sequencing

The DNA of samples was extracted using the E.Z.N.A. ^®^Stool DNA Kit (D4015, Omega, Inc., USA) with beads tubes according to the manufacturer’s instructions. The reagent which was designed to uncover DNA from trace amounts of the sample is effective for the preparation of DNA of most bacteria. Nuclear-free water was used as blank. The NanoDrop 2000 UV–Vis Spectrophotometer (Thermo Scientific, Wilmington, USA) was used to evaluate the concentration and purity of the extracted DNA, and 2% agarose gel electrophoresis was used to determine the quality of the extracted DNA. The polymerase chain reaction (PCR) amplification and bioinformatics analysis were performed at LC-Bio Technology Co., Ltd. (Hang Zhou, China).

Variable regions V3–V4 of the bacterial 16S rRNA gene were amplified with slightly modified versions of primers 341F (5′-CCTACGGGNGGCWGCAG-3′) and 805R (5′-GACTACHVGGGTATCTAATCC-3′) [79]. The 5′ ends of the primers were tagged with specific barcodes per sample and sequencing universal primers.

The 25 ng of template DNA, 12.5 uL of PCR premix and 2.5 uL of each primer were used to PCR amplification. The PCR-grade water was used to adjust the volume until the total volume was 25 mL. The PCR conditions to amplify the prokaryotic 16S fragments consisted of an initial denaturation for 30 s (98 °C); 35 cycles of denaturation for 10 s (98 °C), annealing for 30 s (54 °C/52 °C), extension for 45 s (72 °C); and then final extension for 10 min (72 °C). The PCR products were assessed with 2% agarose gel electrophoresis. In the DNA extraction process, ultrapure water was used to exclude the possibility of false-positive PCR results as a negative control. The PCR products were purified using AMPure XT beads (Beckman Coulter Genomics, Danvers, MA, USA) and quantified using Qubit (Invitrogen, USA). The libraries were sequenced either on 300PE MiSeq plat form and performed for 100 runs.

### Sequencing data analysis

Paired-end reads were assigned to samples according to their unique barcode and truncated by cutting off the barcode and primer sequence and merged with FLASH (v1.2.8) [80]. Quality filtering on the raw tags and high-quality clean tags were carried out under specific filtering conditions to the fqtrim (v0.94) [81]. Chimeric sequences were filtered with Vsearch software (v2.3.4) [82], and the high-quality sequences at a cut off level of 3% were used to assign operational taxonomic units (OTUs) by UPARSE (version 7.1, http://drive5.com/uparse/) [81]. The OTUs were classified using the SILVA database (https://www.arbsilva.de/) with a confidence threshold of 70%. The false discovery rate (FDR)-adjusted Kruskal–Wallis multiple comparisons (*p* < 0.05) were used to detect the bacterial community structure and analyze at the phylum and genus levels [83]. QIIME2 was used to determine the alpha diversity and beta diversity. The Venn diagram was populated according to the common and unique OTUs by R (version 1.6.2). The graphics were drawing with the Omic Studio tools (https://www.omicstudio.cn/tool).

### LC–MS metabolomics processing

The 11 serum samples were analyzed using the LC–MS platform (Thermo, Ultimate 3000LC, Q Exactive). All samples were thawed at 4 °C, each sample (100 µL) was transferred into centrifuge tubes (1.5 mL) and methanol (300 µL) was added to each tube, and mixed for 60 s. Then the sample was centrifuged at 12,000 rpm for 10 min (4 °C) and then transferred into another centrifuge tube (1.5 mL). Samples were concentrated in vaccum and dissolved with 2-chlorobenzalanine methanol solution (150 µL), and filtered through a 0.22 um membrane for LC–MC analysis. 20 µL from each sample was taken to the quality control (QC) samples and the rest of each sample was used for LC–MS detection. Chromatographic separation was performed using a Thermo Vanquish system equipped with an ACQUITY UPLC^®^ HSS T3 column (150 × 2.1 mm, 1.8 µm, Waters) preheated to 40 °C. The samples were injected and maintained at 8 °C for analysis. Gradient elution of analytes was carried out with 0.1% formic acid in water (A) and 0.1% formic acid in acetonitrile (B) at a flow rate of 0.25 mL/min and the following mobile phase (A:B) elution gradient: 2% B for 0–1 min; 2–50% B for 1–9 min; 50%–98% for 9–12 min; 98% B for 12–13.5 min, 13.5–14 min; 98%–100% B for 14–20 min. The rate of sheath gas and auxiliary gas were 30 and 10 arbitrary units, respectively. The spray voltage was 3.8 kV and − 2.5 kV for positive ion mode (ESI+) and negative ion mode (ESI–).

### Metabolomics data and fatty acid profile analysis

The data were transformed to CDF files using Thermo Scientific™ Xcalibur™ (version v3.0). XCMS software (version v.3.4.4) was used for Peak picking, peak alignment, peak filtering, and peak filling. Microsoft Excel was used for retention time (RT), MZ, observations (samples), and peak intensity normalizing. The data were transferred into the SIMCA-P software package. After mean centering and unit variance scaling, principle component analysis (PCA) and (orthogonal) partialleast squares discriminant analysis (O)PLS-DA were used to visualize the metabolic alterations between the grass and concentrate groups. The overall contribution of each variable to the PLS-DA model was based on variable importance in the projection (VIP) ranks and VIP > 1.0 was also considered relevant for group discrimination.

Significant differences in metabolites between the grass and concentrate groups were analyzed involved a combination of (O)PLS-DA and *P* < 0.05 as statistical significance. Differential metabolites were screened using the https://www.i-sanger.com and https://metlin.scripps.edu database. The gplots package in R was used for significant metabolites for expression pattern clustering using [84]. Spearman between samples, Pearson between metabolites, and clustering method for H cluster (complete algorithm) were used for distance calculation algorithms. The metabolic pathways and metabolites set enrichment was analyzed with the Stats package in R and the SciPy package in Python using the MetaboAnalyst 4.0 (https://www.metaboanalyst.ca) [85]. The fatty acids profiles were measured according to the AOAC and Bu et al. methods with a gas chromatography–mass spectrometer 7890B (Agilent, California, United States) [27, 78]. Correlations analysis was assessed by Spearman’s correlation using the pheatmap package in R [86].

### Statistical analysis

Significant differences between the two groups were analyzed using T-tests, with the *p* < 0.05 as statistical significance.

### Supplementary Information

**Additional file 1: Figure S1.** Observed operational taxonomic unit (OTU) line chart.** Figure S2.** Total principal component analysis (PCA)A of the lamb rumen samples corresponding to different feed groups following (**A**) positive and (**B**) negative mode ionization. C, concentrate group; G, grass group.** Table S1.** Ingredients and chemical composition of the experimental diets.** Table S2.** Identification of significant differential metabolites in lamb rumen fluid by comparison of the concentrate and grass groups following positive mode ionization using a VIP threshold of 1 (*P* < 0.05).** Table S3.** Identification of significant differential metabolites in lamb rumen fluid by comparison of the concentrate and grass groups following negative mode ionization using a VIP threshold of 1 (*P* < 0.05).

## Data Availability

The datasets generated for this study are available upon request to the corresponding author.

## Abbreviations

G: Grass group
C: Concentrate group
SEM: Standard error of the mean
OTUs: Operational taxonomic units
PCoA: Principal coordinates analysis
LDA: Linear discrimination analysis
LEfSe: Linear discrimination analysis coupled with effect sizeanalysis
(O)PLS-DA: Orthogonal partial least squares discriminant analysis
