# Supplementary material for: Integrated rumen microbiome and serum metabolome analysis responses to feed type that contribution to meat quality in lambs
Source: Anim Microbiome. 2023 Dec 19;5:65. doi: 10.1186/s42523-023-00288-y (PMC10729572; doi:10.1186/s42523-023-00288-y)
Supplement: Supplementary file 1 — Additional file 1: Figure S1. Observed operational taxonomic unit (OTU) line chart. Figure S2. Total principal component analysis (PCA)A of the lamb rumen samples corresponding to different feed groups following (A) positive and (B) negative mode ionization. C, concentrate group; G, grass group. Table S1. Ingredients and chemical composition of the experimental diets. Table S2. Identification of significant differential metabolites in lamb rumen fluid by comparison of the concentrate and grass groups following positive mode ionization using a VIP threshold of 1 (P < 0.05). Table S3. Identification of significant differential metabolites in lamb rumen fluid by comparison of the concentrate and grass groups following negative mode ionization using a VIP threshold of 1 (P < 0.05). [file 42523_2023_288_MOESM1_ESM.docx]

**Supplementary Table 1** Ingredients and chemical composition of the experimental diets.

| Items | G | C |
| --- | --- | --- |
| Ingredients % |  |  |
| Native grass | 95 | 60 |
| Maize | – | 20 |
| Soybean meal | – | 8 |
| DDGs | – | 4 |
| Wheat bran | – | 3 |
| Salt | 1 | 1 |
| Mineral premix a | 4 | 4 |
| Chemical compositions | |  |
| Dry matter (%) | 91.26 | 92.87 |
| Crude protein (% DM) | 8.53 | 10.04 |
| Ether extract (% DM) | 2.84 | 3.65 |
| Acid detergent fiber (% DM) | 40.12 | 29.43 |
| Neutral detergent fiber (% DM) | 57.47 | 40.25 |

C, concentrate group; G, grass group.


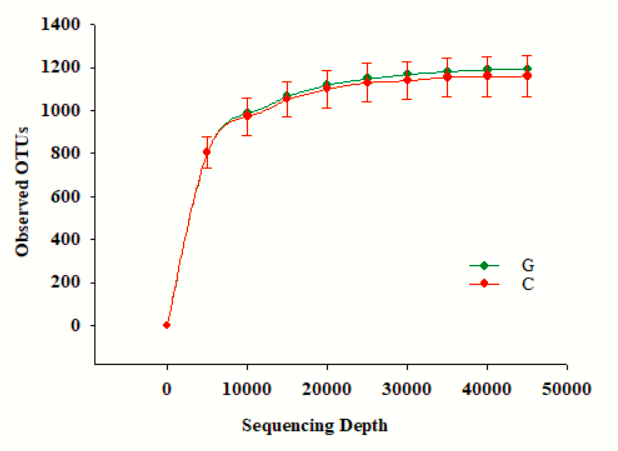


**Supplementary Fig. 1** Observed operational taxonomic unit (OTU) line chart.


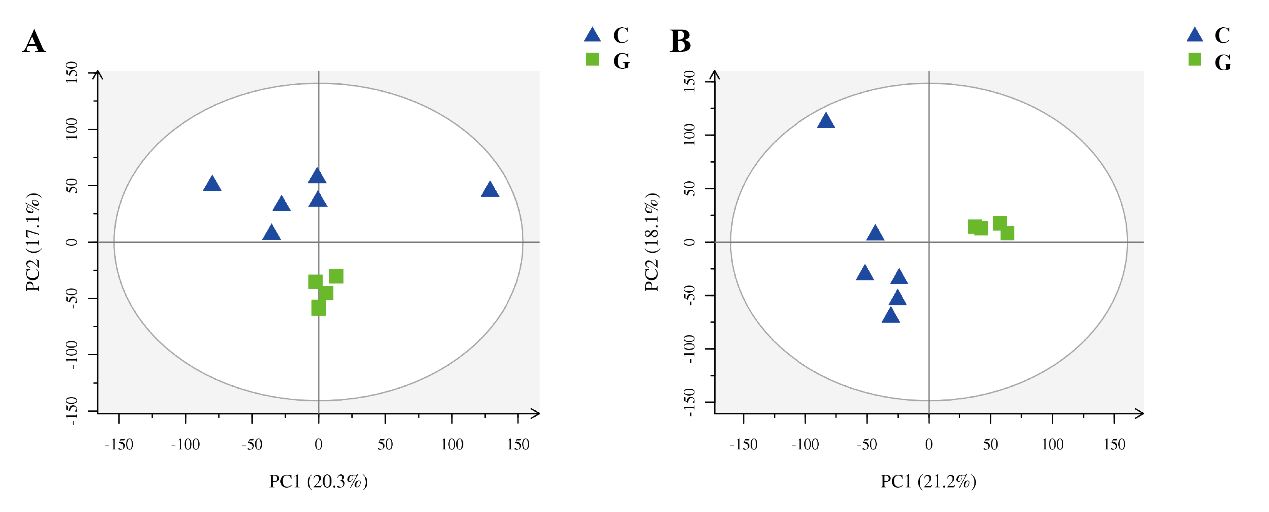


**Supplementary Fig. 2** Total principal component analysis (PCA)A of the lamb rumen samples corresponding to different feed groups following **(A)** positive and **(B)** negative mode ionization. C, concentrate group; G, grass group.

**Supplementary Table 2** Identification of significant differential metabolites in lamb rumen fluid by comparison of the concentrate and grass groups following positive mode ionization using a VIP threshold of 1 (*P*< 0.05).

| Items | VIP | RT (min) | Mass error (ppm) | lon (m/z) | Fold Change | *P*-value | Positive/negative |
| --- | --- | --- | --- | --- | --- | --- | --- |
| Amino acids, peptides, and analogues |  |  |  |  |  |  |  |
| Citrulline | 2.1405 | 1.48 | 1 | 176.1 | 0.63 | <0.05 | ESI+ |
| Pipecolic acid | 2.0076 | 1.66 | 1 | 130.09 | 0.31 | <0.05 | ESI+ |
| Ectoine | 1.9205 | 1.78 | 0 | 143.08 | 0.49 | <0.05 | ESI+ |
| L-Serine | 1.9665 | 1.44 | 5 | 106.05 | 0.61 | <0.05 | ESI+ |
| L-Glutamine | 1.8764 | 1.45 | 1 | 147.08 | 0.72 | <0.05 | ESI+ |
| L-Isoleucine | 1.7615 | 3.43 | 0 | 132.1 | 0.61 | <0.05 | ESI+ |
| L-Tyrosine | 1.6954 | 3.37 | 0 | 182.08 | 0.68 | <0.05 | ESI+ |
| L-Arginine | 1.5783 | 5.31 | 0 | 245.15 | 0.75 | <0.05 | ESI+ |
| Ornithine | 1.5891 | 1.65 | 2 | 133.1 | 0.71 | <0.05 | ESI+ |
| Pyroglutamic acid | 1.5452 | 2.67 | 2 | 130.05 | 0.58 | <0.05 | ESI+ |
| Bile acids, alcohols and derivatives |  |  |  |  |  |  |  |
| Glycocholic acid | 1.8148 | 11.76 | 1 | 466.32 | 0.05 | <0.05 | ESI+ |
| 25-Hydroxycholesterol | 1.8099 | 6.99 | 2 | 402.21 | 3.47 | <0.05 | ESI+ |
| Lithocholic acid | 1.7694 | 13.63 | 1 | 376.31 | 2.74 | <0.05 | ESI+ |
| Purines and purine derivatives |  |  |  |  |  |  |  |
| Theophylline | 1.9795 | 7.73 | 14 | 181.07 | 0.51 | <0.05 | ESI+ |
| 3-Methylxanthine | 1.6239 | 3.36 | 5 | 167.06 | 0.56 | <0.05 | ESI+ |
| Phosphate esters |  |  |  |  |  |  |  |
| O-Phosphoethanolamine | 1.6538 | 0.53 | 1 | 141.96 | 1.39 | <0.05 | ESI+ |
| Phosphoglycolic acid | 1.7118 | 16.87 | 6 | 155.99 | 0.76 | <0.05 | ESI+ |
| Fatty acids and conjugates |  |  |  |  |  |  |  |
| Glutarate semialdehyde | 2.0273 | 1.56 | 12 | 116.05 | 0.48 | <0.05 | ESI+ |
| Benzoic acids and derivatives |  |  |  |  |  |  |  |
| p-Aminobenzoic acid | 2.1377 | 1.57 | 0 | 138.06 | 0.34 | <0.05 | ESI+ |
| Linoleic acids derivatives |  |  |  |  |  |  |  |
| Methyl jasmonate | 1.6565 | 17.12 | 3 | 223.99 | 0.84 | <0.05 | ESI+ |
| Others |  |  |  |  |  |  |  |
| Methylmalonic acid | 2.0962 | 11.80 | 5 | 101.07 | 0.37 | <0.05 | ESI+ |
| (+)-7-Isojasmonic acid | 1.8656 | 12.39 | 0 | 211.13 | 3.11 | <0.05 | ESI+ |
| (R) 2,3-Dihydroxy-3-methylvalerate | 1.8470 | 1.45 | 1 | 148.08 | 0.69 | <0.05 | ESI+ |
| 1-Hexadecanol | 1.8042 | 6.30 | 2 | 243.18 | 4.77 | <0.05 | ESI+ |
| Ketoleucine | 1.7912 | 17.00 | 3 | 130.97 | 0.74 | <0.05 | ESI+ |
| Isocitric acid | 1.7455 | 1.71 | 2 | 192.03 | 4.25 | <0.05 | ESI+ |
| Taurine | 1.7717 | 1.45 | 1 | 126.02 | 1.96 | <0.05 | ESI+ |
| Norepinephrine | 1.7754 | 10.63 | 1 | 169.09 | 2.27 | <0.05 | ESI+ |
| 2-Phenylacetamide | 1.6692 | 3.37 | 2 | 136.08 | 0.69 | <0.05 | ESI+ |
| Pyrrolidonecarboxylic acid | 1.6389 | 1.74 | 2 | 130.05 | 0.77 | <0.05 | ESI+ |
| Indolepyruvate | 1.6208 | 3.37 | 0 | 204.06 | 0.69 | <0.05 | ESI+ |
| Betaine aldehyde | 1.5845 | 18.78 | 1 | 102.09 | 0.66 | <0.05 | ESI+ |
| Gentamicin C1a | 1.5230 | 7.58 | 5 | 432.28 | 0.60 | <0.05 | ESI+ |
| 2-Aminophenol | 1.5029 | 1.03 | 3 | 110.06 | 0.69 | <0.05 | ESI+ |
| Indoleglycerol phosphate | 1.4935 | 1.46 | 1 | 288.07 | 2.03 | <0.05 | ESI+ |

RT, retention time; fold change, concentrate group vs. forage group. Mass Error in ppm, the difference between a theoretical m/z and an experimentally observed m/z. ESI+, positive ion mode.

**Supplementary Table 3** Identification of significant differential metabolites in lamb rumen fluid by comparison of the concentrate and grass groups following negative mode ionization using a VIP threshold of 1 (*P*< 0.05).

| Items | VIP | RT (min) | Mass error (ppm) | lon (m/z) | Fold Change | *P*-value | Positive/negative |
| --- | --- | --- | --- | --- | --- | --- | --- |
| Amino acids, peptides, and analogues |  |  |  |  |  |  |  |
| L-Lysine | 1.6689 | 1.59 | 0 | 147..11 | 0.59 | <0.05 | ESI- |
| N6-Acetyl-L-lysine | 1.6350 | 2.47 | 5 | 187.11 | 0.68 | <0.05 | ESI- |
| Benzoic acids and derivatives |  |  |  |  |  |  |  |
| Hippuric acid | 1.8551 | 5.67 | 1 | 179.05 | 0.46 | <0.05 | ESI- |
| Protocatechuic acid | 1.7224 | 7.35 | 7 | 153.02 | 0.30 | <0.05 | ESI- |
| Linoleic acids derivatives |  |  |  |  |  |  |  |
| Jasmonic acid | 1.5831 | 10.66 | 4 | 209.12 | 2.72 | <0.05 | ESI- |
| Fatty acids and conjugates |  |  |  |  |  |  |  |
| Capric acid | 1.6893 | 13.44 | 7 | 171.14 | 1.37 | <0.05 | ESI- |
| Others |  |  |  |  |  |  |  |
| 1,4-Dihydroxy-2-naphthoate | 2.0919 | 10.02 | 4 | 203.03 | 0.16 | <0.05 | ESI- |
| Lipoxin A4 | 1.9751 | 13.11 | 4 | 351.22 | 0.26 | <0.05 | ESI- |
| Glycochenodeoxycholic acid | 1.8100 | 11.23 | 3 | 448.31 | 0.19 | <0.05 | ESI- |
| α-dimorphecolic acid | 1.7136 | 13.97 | 0 | 295.23 | 0.47 | <0.05 | ESI- |
| O-Acetylcarnitine | 1.6688 | 1.42 | 1 | 238.93 | 0.92 | <0.05 | ESI- |
| 3-(2-Hydroxyphenyl) propanoic acid | 1.6257 | 6.96 | 1 | 165.05 | 0.17 | <0.05 | ESI- |
| trans-Cinnamate | 1.6169 | 2.63 | 1 | 146.96 | 0.62 | <0.05 | ESI- |
| 3-Hydroxyphenylacetic acid | 1.5612 | 6.07 | 4 | 151.04 | 0.55 | <0.05 | ESI- |
| Gluconic acid | 1.5553 | 1.36 | 1 | 195.05 | 1.81 | <0.05 | ESI- |
| β-Alanyl-L-lysine | 1.5437 | 1.59 | 2 | 198.12 | 0.21 | <0.05 | ESI- |
| dTMP | 1.5418 | 13.53 | 11 | 321.05 | 1.07 | <0.05 | ESI- |
| dGMP | 1.4740 | 14.95 | 1 | 347.17 | 0.30 | <0.05 | ESI- |
| Pyruvic acid | 1.4149 | 1.53 | 4 | 88.01 | 1.61 | <0.05 | ESI- |

RT, retention time; fold change, concentrate group vs. forage group. Mass Error in ppm, the difference between a theoretical m/z and an experimentally observed m/z. ESI−, negative ion mode.
